# The relative efficacy of nine osteoporosis medications for reducing the rate of fractures in post-menopausal women

**DOI:** 10.1186/1471-2474-12-209

**Published:** 2011-09-26

**Authors:** Robert B Hopkins, Ron Goeree, Eleanor Pullenayegum, Jonathan D Adachi, Alexandra Papaioannou, Feng Xie, Lehana Thabane

**Affiliations:** 1 Department of Clinical Epidemiology and Biostatistics, Faculty of Health Science, McMaster University, Hamilton, Ontario, Canada; 2 Programs for Assessment of Technology in Health, St Joseph's Healthcare-Hamilton, Hamilton, Ontario, Canada; 3 Centre for Evaluation of Medicines, Hamilton, Ontario, Canada; 4 Biostatistics Unit, St Joseph's Healthcare-Hamilton, Hamilton, Ontario, Canada; 5 Department of Medicine, McMaster University, Hamilton, Ontario, Canada

## Abstract

**Background:**

In the absence of head-to-head trials, indirect comparisons of randomized placebo-controlled trials may provide a viable option to assess relative efficacy. The purpose was to estimate the relative efficacy of reduction of fractures in post-menopausal women, and to assess robustness of the results.

**Methods:**

A systematic literature review of multiple databases identified randomized placebo-controlled trials with nine drugs for post-menopausal women. Odds ratio and 95% credibility intervals for the rates of hip, non-vertebral, vertebral, and wrist fractures for each drug and between drugs were derived using a Bayesian approach. A drug was ranked as the most efficacious if it had the highest posterior odds ratio, or had the highest effect size.

**Results:**

30 studies including 59,209 patients reported fracture rates for nine drugs: alendronate (6 studies), denosumab (1 study), etidronate (8 studies), ibandronate (4 studies), raloxifene (1 study), risedronate (7 studies), strontium (2 study), teriparatide (1 study), and zoledronic acid (1 study). The drugs with the highest probability of reducing non-vertebral fractures was etidronate and teriparatide while the drugs with the highest probability of reducing vertebral, hip or wrist fractures were teriparatide, zoledronic acid and denosumab. The drugs with the largest effect size for vertebral fractures were zoledronic acid, teriparatide and denosumab, while the drugs with the highest effect size for non-vertebral, hip or wrist fractures were alendronate or risedronate. Estimates were consistent between Bayesian and classical approaches.

**Conclusion:**

Teriparatide, zoledronic acid and denosumab have the highest probabilities of being most efficacious for non-vertebral and vertebral fractures, and having the greatest effect sizes. The estimates from indirect comparisons were robust to differences in methodology.

## Background

Osteoporosis defined by low Bone Mineral Density (BMD) (i.e., 2.5 standard deviations below peak gender specific BMD), is a progressive disease with high prevalence affecting 1 in 3 women and 1 in 8 men by the time they reach 90 years of age [1]. The major concern with low BMD is the high risk of fractures to non-vertebral bones such as the wrist or to the hip. A hip fracture may require extended hospital stay, surgical repair and rehabilitation therapy, and is associated with increased risk of death [2]. In addition, osteoporosis can lead to vertebral fractures which are identified by clinical assessment through decreased patient height (i.e., stooped posture) or with compressed spinal vertebra that can be radiologically assessed [3].

To reduce the risk of fractures due to osteoporosis, drugs have been introduced to reduce the rate of bone loss and to increase the strength of the bones. The first bisphosphonate available in Canada was etidronate in 1995 followed by alendronate in 1998. In 2005, alendronate became generic, which introduced a large increase in the uptake of these drugs. In Canada in 2010 about 9% of the population age 50 years and over were receiving an osteoporosis drug. These drugs include the bisphosphonates (alendronate, etidronate, risedronate or ibandronate), Selective Estrogen Receptor Modulators (raloxifene) and anabolic agents (teriparatide). All of these have shown to be effective in reducing the rate of fractures relative to placebo [4]. Recent additions to pharmacotherapy for osteoporosis include denosumab, strontium and zoledronic acid. Accordingly, it would be clinically important to know an estimate of the relative treatment efficacy or ranking of the most efficacious drugs. A major gap in the evidence to identify the most efficacious drugs is the lack of randomized active-controlled trials, i.e., direct treatment comparison (DTC) evidence [4].

DTC evidence for osteoporosis is absent because such later stage III trials are more complex, expensive, and require larger sample sizes than earlier phase II randomized placebo-controlled trials (RPCT) [5]. Meanwhile, osteoporosis drugs have been approved for use or listed under reimbursement formularies based on RPCT evidence, even though there are skeptics on the benefits of RPCTs for estimating relative efficacy compared to currently available drugs and that RPCTs are unethical. In a recent New England Journal of Medicine debate, Stein [6] argued that RPCTs are unethical because of the withholding of proven therapies in the placebo allocation, while Rosen [7] argued that the therapies are only proven in high risk patients (prior fracture, BMD < -3, or higher fracture risk assessment) and the inclusion criteria that possess true equipoise should only include individuals who are at low risk or are non-responsive to mild therapies. However in Canada the Tri-Council Policy Statement on the Ethical Conduct for Research Involving Humans suggest that RPCTs are acceptable to establish existence of effect and adverse events of drugs with new pharmacological mechanisms [8]. In the absence of DTC evidence, indirect treatment comparisons (ITC) might be a promising technique that allows the synthesis of available RPCT evidence to make a suggestion on the effect of DTC [9]. The theoretical foundations of the ITC method were provided in 1997 by Bucher [10] for the pair wise division of odds ratios to produce a common odds ratio thereafter referred to as the Bucher Method (i.e., for 2 drugs A and C and placebo B, the odds ratio of A/B divided by odds ratio C/B produces an odds ratio of A/C). While DTC is the highest level of clinical evidence, there exists the rationale to use ITC analysis where DTC is absent and not likely to be forthcoming [10]. Even if DTC evidence was available, ITC evidence based on other trials may be useful because of differences in patient characteristics and study characteristics such as length of follow-up [11].

In the absence of DTC evidence for osteoporosis drugs, two ITC analyses have been conducted to assess the relative efficacy at reducing the rates of fractures in post-menopausal women [12,13]. The first using a Bayesian analysis that looked at seven studies including four drugs zoledronic acid (1 study), alendronate (3 studies), ibandronate (1 study) and risedronate (2 studies). this indicated that zoledronic acid had the highest efficacy in preventing vertebral fractures [12]. The second and more comprehensive analysis included eight RPCTs which was an update involving the above four medications adding etidronate (1 study). Of the five medications analyzed zoledronic acid had the highest efficacy in preventing vertebral and hip fractures while risedronate had the highest efficacy in reducing non-vertebral non-hip fractures [13].

We believe we can build on this pioneering work. First, nine drugs are currently available in Canada, European or the United States for use with osteoporosis. The nine drugs include the above five (zoledronic acid, alendronate, ibandronate, and risedronate) in the recent ITC analysis plus four more drugs that were not previously included (denosumab, raloxifene, strontium, and teriparatide). In addition, there are key differences in patient characteristics across the studies such as age, BMD, and fracture history. Further adjustment for these factors might affect estimates of the relative efficacy between treatments.

The purpose of this paper is to build on the previous estimation of relative efficacy between osteoporosis drugs for the prevention of fractures. First, we update the literature on osteoporosis drugs to include recent additions in pharmacotherapy and recent RPCTs by conducting a multiple database systematic literature review. Second, we estimate the relative efficacy of reducing fractures of each drug versus placebo and between the drugs with Bayesian ITC analysis. Third, we conduct the ITC analysis using Bucher's method, a classical analysis approach. Finally, we estimated the relative efficacy after adjustment for baseline patient characteristics.

## Methods

### Literature Search

An electronic search of the following databases restricted to English was conducted from January 1990 to October 2009, and the search was continually updated by alerts until September 2010: EMBASE, Medline, Medline in Process, and Cochrane Database for Systematic Reviews, Evidence Based Reviews-American College of Physicians Journal, National Health Service (NHS) Database of Assessment of Reviews and Effectiveness (DARE), CINAHL. Specific searches were developed for each database with the aid of professional librarian and were based on MeSH headings and keywords: osteoporosis, fractures, and bones. Methodological filters for randomization were applied to Medline and EMBASE (see additional file 1 for the search strategy). We also conducted a bibliographic search on each article that was identified. Following the literature searches, all citations were incorporated into Reference Manager citation database software [14] and duplicates were identified and removed.

The inclusion criteria was that each article must have; 1) one of the nine osteoporosis drugs: alendronate, denosumab, etidronate, ibandronate, raloxifene, risedronate, strontium, teriparatide, or zoledronic acid, 2) have a RPCT design, 3) have only post-menopausal women as an inclusion criterion, and 4) report the rate of fractures as a primary or secondary outcome. Studies were excluded if they were studies that combined different trials, were subgroup analysis, or the outcomes were not fractures such as BMD.

### Selection of trials for inclusion and data abstraction

At the first level of screening of the publications, the titles and abstracts of the citations that were obtained from the search strategy were reviewed for relevance and inclusion for full-text review. Of these articles passing to level two, the articles were reviewed as full text for relevance. After inclusion, data was abstracted to pre-specified abstraction forms and then entered into Microsoft Excel and the Bayesian meta-analysis software WinBUGS (**B**ayesian inference **U**sing **G**ibbs **S**ampling for Windows) [15]. For the literature retrieved based on the targeted review for systematic reviews and meta-analysis, the same 4 inclusion criteria applied. Literature screening was conducted by two independent reviewers, with consensus reached on all discrepancies.

### Outcomes

The main outcomes were the rates of vertebral, non-vertebral, hip and wrist fractures. In addition, study characteristics (country, numbers of study centres, and patient follow up in years) and baseline patient characteristics (age in years, years since menopause, BMD of the hip reported as g/cm^2^, and history of fractures) were abstracted. Data abstraction was verified by a second independent reviewer. For each outcome, the unadjusted odds ratio for each drug versus placebo were estimate along with its 95% CrI. In addition, the adjusted for fracture between each drug comparator was estimated along with its 95% CrI (i.e.,  Odds ratio of A/B = odd ratio (A/B) divided by odds ratio of C/B). 

### Primary Statistical Analysis: Bayesian ITC estimate of relative efficacy versus placebo and other drugs

ITC was conducted for the unadjusted analysis using Bayesian methods in WinBUGS software version 1.4.3 [15], which performs Bayesian analysis using Markov Chain Monte Carlo methods (see additional file 2 for software code). We reported the analysis according to the Reporting Of Bayes used in clinical STudies (ROBUST) criteria [16]. The outcome estimated was the mean and the 95% credibility interval of the posterior distribution of the odds ratio of the rate of fracture versus placebo and other drugs, for each fracture. For the Bayesian analysis, priors were predefined for the mean log odds ratio as a normal distribution with mean zero, and precision 0.001 representing weak prior information. Weak priors were chosen so that the final estimates for odds ratios are driven by the data, and not by any assumption made. For each outcome, we performed 100,000 simulations discarding the first 50,000 simulations to allow burn-in; two chains were run simultaneously. Convergence was assessed using all of the Geweke, Raftery-Lewis, Gelman-Rubin and Heidelberger-Welch tests. To make a comparison of all drugs to each in order to determine the most effect efficacious drug, the proportion of Markov chain iterations in which a drug had the highest odds ratio represented the probability of that drug being ranked the most efficacious. In addition, the effect size was estimated for each drug versus placebo, where effect size was defined as the ratio of the odds ratio for fracture of placebo versus drug divided by the standard error of the estimate of the odds ratio. A higher effect size indicates the drug has lower odds for fractures than placebo and/or that the standard error is small. Software code for WinBUGS is provided in appendix 2.

### Assessing robustness: homogeneity and consistency of evidence

A number of steps were taken to assess the integrity of the ITC analysis [10,17-20]. The assessments included; 1) assessing homogeneity in meta-analysis of each comparator and across comparators, and 2) checking the consistency of the ITC analysis between Bayesian and classical software, and 3) checking the consistency of the ITC analysis to DTC if available. If there is homogeneity within drugs and across drugs, and the ITC evidence is consistent across methodologies or with DTC evidence, then the ITC evidence in considered strong and free of bias [19].

Homogeneity with each drug and across each drug was assessed with Review Manager 5 software [21]. Heterogeneity was assessed with I^2 ^with greater than 50% being moderate heterogeneity and greater than 70% being considerable heterogeneity as suggested by the Cochrane Handbook of Systematic Reviews [22]. Consistency of evidence was assessed by comparing the results of the Bayesian analysis to free software specifically created for ITC analysis [23]. This software package for ITC was released by the Canadian Agency for Drugs and Technologies in Health (CADTH) [24], a national agency in Canada that provides evidence based decisions and associated services for the national and provincial level governments. Checking consistency of ITC evidence versus DTC evidence was conducted by a search for meta-analysis of DTC evidence.

### Adjustment for difference in baseline characteristics

Lastly, we checked whether differences in patient characteristics across drugs contributed to the relative efficacy estimates in the ITC analysis. We estimated the odds ratios for fracture reduction with classical meta-analysis with meta-regression with the log of the odds ratio as the dependent variable, and dummy variables were added for each of the drugs. Following the unadjusted results, we adjusted the ITC estimates with meta-regression to include the age in years, BMD in g\cm^2^, percent of subjects with history of a vertebral fracture. Meta-regression was conducted with STATA version 11.0 using the command *metareg*.

## Results

**Figure 1:**
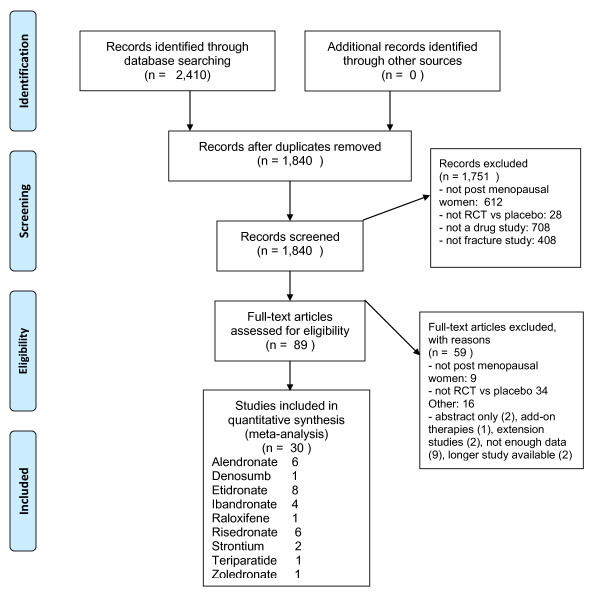
**PRISMA Flow Diagram describing selection process for included studies**. PRISMA (Preferred Reporting Items for Systematic Reviews and Meta-Analyses) Flow Diagram describing selection process for included studies.

Based on the literature review, 30 RPCTs that investigated the effect of drugs on the rate of fractures were identified. The results of the screening process are provided in the PRISMA diagram [25] in Figure 1 and the descriptions of the included studies are presented in Table 1. For the 9 drugs, 6 studies were for alendronate [26-31], 1 study for denosumab [32], 8 studies for etidronate [33-40], 4 studies for ibandronate [41-44], 1 study for raloxifene [45], 6 studies for risedronate [46-51], 2 studies for strontium [52,53], 1 for teriparatide [54], and 1 for zoledronic Acid [55]. The participants in the studies included 59,209 patients. The participants had a mean age ranging in studies between 52 and 72 years of age, years since menopause ranged from 2.7 to 31.9 years, and the study durations were from 1 to 4 years. Baseline BMD in the hip ranged from 0.28 to 1.08 and the percentage of participants that had previous vertebral fractures were from 0% to 100%.

**Table 1:** Description of Study and Baseline Characteristics for Included Studies

Drug	Author Year	Study Duration (years)	Country/Region	Number of Centres	Age (yrs) Mean(SD)	Years Menopause Inclusion Criteria	Years Since Menopause Mean (SD)	BMD Hip g/cm^2^ Mean (SD)	Prior Vertebral Fracture %
Alendronate	Ascott Evans 2003	1	International	18	67.3 (6.6)	3	11.5 (7.3)	nr	0

Alendronate	Black 1996	3	North America	11	71.0 (5.6)	2	NR (NR)	0.57 (0.07)	100

Alendronate	Cummings 1998	4	North America	11	67.6 (6.1)	2	NR (NR)	0.84 (0.13)	0

Alendronate	Greenspan 1998	2.5	North America	1	70.0 (4.6)	NR	NR (NR)	0.57 (0.11)	NR

Alendronate	Liberman 1995	3	International	NR	64.0 (7.0)	5	16.5 (NR)	0.71 (NR)	21

Alendronate	Pols 1999	1	International	153	62.8 (7.4)	3	15.9 (1.5)	0.72 (0.08)	NR

Denosumab	Cummings 2009	4	North America	11	67.7 (6.6)	2	NR (NR)	0.84 (0.13)	0

Etidronate	Lyritis 1997	4	Europe	1	72.0 (0.4)	NR	25.8 (1.7)	0.57 (NR)	100

Etidronate	Meunier 1997	4	Europe	1	52.7 (4.0)	0.5	2.4 91.8)	0.90 (NR)	NR

Etidronate	Montesorri 1997	3	Europe	2	62.5 (6.2)	1	14.9 (6.1)	0.67 (NR)	29

Etidronate	Pacifici 1988	2	U.S.A	1	61.0 (7.8)	NR	13.8 (9.5)	0.79 (0.26)	100

Etidronate	Pouilles 1997	2	Europe	7	53.8 (3.1)	0.5	2.6 (1.4)	0.96 (NR)	NR

Etidronate	Storm 1990	3	Europe	1	68.3 (7.3)	NR	21.6 (10.2)	0.25 (0.07)	100

Etidronate	Watts 1990	2	U.S.A	7	65.1 (13.0)	1	17.9 (16.5)	0.86 (NR)	100

Etidronate	Wimalawansa 1998	4	NR	NR	64.9 (7.8)	NR	15.1 (6.8)	0.83 (NR)	100

Ibandronate	Chesnut 2004	3	Europe, U.S.A	73	69.0 (11.0)	5	21 (20.8)	0.78 (NR)	93

Ibandronate	Ravn 2002	1	Europe	1	64.5 (5.9)	10	NR (NR)	0.87 (0.13)	28

Ibandronate	Adami 2004	1	Europe	NR	65.9 (4.5)	5	17.9 (4.0)	0.77 (0.09)	45

Ibandronate	Recker 2004	3	Europe	NR	67.0 (5.1)	5	NR (NR)	0.80 (0.11)	54

Raloxifene	Ettinger 1999	3	International	180	66.1 (6.9)	2	18.6 (7.9)	0.58 (NR)	38

Risedronate	Fogelman 2000	2	Europe	13	64.7 (7.2)	1	17.7 (9.4)	0.74 (0.08)	30

Risedronate	Harris 1999	3	North America	110	69.0 (7.3)	5	24.0 (9.9)	0.83 (0.16)	81

Risedronate	Hooper 2005	2	Australia	11	52.6 (3.3)	0.5	3.9 (5.6)	1.08 (0.12)	18.3

Risedronate	McClung 2001	3	International	183	78.0 (9.7)	NR	31.8 (19.3)	NR (NR)	42

Risedronate	Mortenson 1998	2	International	2	51.2 (3.8)	0.5	2.7 91.7)	0.94 (0.11)	NR

Risedronate	Reginster 2000	3	Europe, Australia	80	71.0 (7.0)	5	24.4 (8.5)	0.79 (0.15)	100

Strontium	Meunier 2004	3	Europe, International	72	69.3 (7.3)	5	43.7 (8.7)	0.68 (0.11)	100

Strontium	Reginster 2008	3.5	International	75	76.7 (5.0)	0	28.4 (7.4)	0.55 (NR)	33.5

Teriparatide	Neer 2001	2	International	99	69.0 (7.0)	5	21.0 (8.0)	0.82 (0.17)	100

Zoledronate	Black 2007	3	U.S.A, Europe	60	73 (5.4)	0	NR (NR)	0.65 (0.91)	36.7

NR: Not reported. BMD: Bone Mineral Density. SD: Standard deviation. U.S.A: United States of America

### Bayesian ITC estimate of relative efficacy versus placebo and other drugs

The estimates of relative efficacy of each drug versus placebo in the Bayesian meta-analysis is reported in table 2. For non-vertebral fractures, only alendronate OR = 0.81 (95%CrI: 0.66, 0.96) and risedronate OR = 0.77 (95%CrI: 0.60, 0.91) had significant reduction. Etidronate had the highest probability of being most efficacious (0.41) along with teriparatide (0.41). All other drugs had less than 0.10 probability of being most efficacious. However, the drugs with the highest effect size were Risedronate (16.4) and Alendronate (16.1), but these effect sizes were smaller than the effect sizes for vertebral fractures. Based on the probabilities of being most efficacious, etidronate and zoledronic acid are the most efficacious drugs. However since etidronate does not have significant effect versus placebo, teriparatide is the most efficacious drug. In the ITC head-to-head analysis (Table 3) there is not enough evidence to detect differences in efficacy between any of the drugs for non-vertebral fractures, although teriparatide, zoledronic acid and denosumab have the lowest numbers need to treat to prevent a non-vertebral fracture versus the other drugs.

**Table 2:** Odds Ratio for Fracture, Indirect Treatment Comparison Results of Drug versus Placebo Classical and Bayesian analysis

Classical analysis	Non-vertebral fracture			Vertebral fracture			Hip fracture			Wrist fracture			
**Drug vs placebo**	**OR (95% Cr I)**	**Placebo** **rate**		**OR (95% Cr I)**	**Placebo** **rate**		**OR (95% Cr I)**	**Placebo** **rate**		**OR (95% Cr I)**	**Placebo** **rate**		

Alendronate	0.80 (0.68, 0.95)	11.1%		0.51 (0.40, 0.63)	6.7%		0.62 (0.40, 0.96)	1.1%		0.44 (0.30, 0.67)	3.0%		

Denosumab	0.80 (0.67, 0.96)	7.5%		0.31 (0.24, 0.40)	7.2%		0.60 (0.37, 0.98)	1.1%		NR	NR		

Etidronate	0.64 (0.31, 1.32)	11.5%		0.59 (0.32, 1.10)	9.7%		0.60 (0.14, 2.66)	2.1%		1.19 (0.37, 3.80)	2.2%		

Ibandronate	0.88 (0.71, 1.10)	7.5%		0.49 (0.32, 0.73)	7.5%		NR	NR		NR	NR		

Raloxifene	0.91 (0.77, 1.07)	9.3%		0.63 (0.50, 0.78)	10.1%		1.12 (0.64, 1.95)	0.7%		0.88 (0.67, 1.15)	3.3%		

Risedronate	0.79 (0.69, 0.89)	10.1%		0.59 (0.47, 0.75)	13.3%		0.74 (0.58, 0.94)	2.8%		0.71 (0.56, 0.89)	3.4%		

Strontium	0.85 (0.74 (0.98)	14.7%		0.58 (0.50, 0.67)	21.7%		0.66 (1.19)	4.0%		1.59 (1.12, 2.27)	3.2%		

Teriparatide	0.62 (0.40, 0.97)	9.7%		0.31 (0.19, 0.52)	14.3%		0.50 (0.09, 2.75)	0.7%		0.50 (0.09, 2.75)	2.4%		

Zoledronic Acid	0.74 (0.63, 0.86)	10.0%		0.28 (0.22, 0.35)	10.9%		0.59 (0.83)	2.3%		NR	NR		

All drugs vs placebo	0.81 (0.77, 0.86)	10.5%		0.49 (0.41, 0.58)	11.0%		0.73 (0.63, 0.84)	1.9%		0.82 (0.71, 0.94)	3.1%		

**Bayesian analysis**	**Non-vertebral fracture**			**Vertebral fracture**			**Hip fracture**			**Wrist fracture**			

**Drug vs placebo**	**OR (95% Cr I)**	**Prob**	Effect size	**OR (95% Cr I)**	Prob	Effect size	**OR (95% Cr I)**	**Prob**	Effect size	**OR (95% Cr I)**		**Prob**	Effect size

Alendronate	0.81 (0.66, 0.96)	0.01	16.1	0.51 (0.37, 0.68)	< 0.01	25.3	0.59 (0.29, 0.99)	0.10	9.49	0.93 (0.30, 2.64)		0.10	1.80

Denosumab	0.80 (0.60, 1.06)	0.03	10.7	0.31 (0.21, 0.44)	0.20	53.6	0.67 (0.24, 1.47)	0.12	4.76	NR		NR	NR

Etidronate	0.64 (0.31, 1.27)	0.42	6.4	0.61 (0.29, 1.08	0.01	8.3	1.02 (0.12, 3.91)	0.19	1.01	2.42 (0.25, 10.54)		0.06	0.16

Ibandronate	0.90 (0.69, 1.16)	< 0.01	9.3	0.50 (0.29, 0.78)	0.01	16.1	NR	NR	NR	NR		NR	NR

Raloxifene	0.91 (0.69, 1.20)	< 0.01	8.4	0.63 (0.43, 0.90)	0.00	13.4	1.29 (0.45, 2.88)	0.01	1.25	1.76 (0.09, 8.22)		0.15	0.27

Risedronate	0.77 (0.60, 0.91)	0.04	16.4	0.60 (0.45, 0.79)	0.00	19.3	0.78 (0.44, 1.32)	0.01	5.71	0.91 (0.13, 3.27)		0.22	1.37

Strontium	0.86 (0.69, 1.07)	< 0.01	12.0	0.59 (0.45, 0.76)	< 0.01	21.8	0.98 (0.39, 2.01)	0.01	2.47	3.25 (0.17, 14.89)		0.06	0.08

Teriparatide	0.62 (0.38, 1.02)	0.41	9.9	0.32 (0.17, 0.57)	0.30	29.8	0.71 (0.04, 2.90)	0.44	1.93	1.23 (0.05, 5.64)		0.41	0.57

Zoledronic Acid	0.74 (0.56, 0.97)	0.08	12.9	0.28 (0.19, 0.40)	0.40	66.2	0.65 (0.25, 1.34)	0.11	5.53	NR		NR	NR

OR (95% Cr I): Odds ratio (95% Credibility Interval). Prob: probability of that drug being most efficacious.

Effect size evaluated as Odds ratio divided by corresponding standard error. NR: Not reported.

**Table 3:** Odds Ratio for Fracture, Indirect Treatment Comparison between drugs (Bayesian analysis)

	Non-vertebral fracture		Vertebral fracture		Hip fracture		Wrist fracture	
	**OR (95% CrI)**	**NNT**	**OR (95% CrI)**	**NNT**	**OR (95% CrI)**	**NNT**	**OR (95% CrI)**	**NNT**

Denosumab vs Alendronate	0.99 (0.72, 1.42)	1,063	0.63 (0.38, 0.97)	26	1.30 (0.38, 3.35)	-180	NR	NR

Denosumab vs Etidronate	1.26 (0.59, 2.69)	-42	0.58 (0.26, 1.15)	23	1.43 (0.13, 5.97)	-126	NR	NR

Denosumab vs Ibandronate	0.89 (0.61, 1.31)	96	0.67 (0.35, 1.19)	30	NR	NR	NR	NR

Denosumab vs Raloxifene	0.87 (0.59, 1.30)	81	0.51 (0.29, 0.83)	20	0.71 (0.14, 1.89)	184	NR	NR

Denosumab vs Risedronate	1.04 (0.76, 1.54)	-267	0.53 (0.32, 0.82)	21	0.94 (0.27, 2.24)	893	NR	NR

Denosumab vs Teriparatide	1.29 (0.73, 2.26)	-38	1.06 (0.50, 1.99)	-169	3.24 (0.17, 16.89)	-25	NR	NR

Denosumab vs Zoledronic Acid	1.08 (0.73, 1.62)	-134	1.16 (0.66, 1.88)	-65	1.36 (0.30, 3.48)	-150	NR	-14

Etidronate vs Alendronate	0.79 (0.38, 1.61)	50	1.22 (0.54, 2.28)	-48	1.91 (0.20, 7.43)	-60	3.48 (0.22, 16.27)	NR

Ibandronate vs Alendronate	1.13 (0.82, 1.60)	-83	1.00 (0.54, 1.69)	20,428	NR	NR	NR	NR

Ibandronate vs Etidronate	1.44 (0.68, 3.06)	-25	0.92 (0.37, 1.95)	121	NR	NR	NR	-22

Raloxifene vs Alendronate	1.12 (0.82, 1.55)	-90	1.28 (0.78, 1.98)	-38	2.47 (0.71, 6.55)	-38	2.60 (0.08, 11.84)	-39

Raloxifene vs Etidronate	1.41 (0.68, 2.96)	-27	1.17 (0.53, 2.29)	-62	2.76 (0.24, 11.66)	-32	1.87 (0.03, 9.82)	NR

Raloxifene vs Ibandronate	1.02 (0.70, 1.49)	-533	1.36 (0.71, 2.38)	-29	NR	NR	NR	-108

Risedronate vs Alendronate	0.95 (0.71, 1.23)	212	1.21 (0.79, 1.79)	-50	1.47 (0.62, 3.31)	-115	1.31 (0.10, 5.21)	3,328

Risedronate vs Etidronate	1.19 (0.57, 2.49)	-57	1.11 (0.52, 2.18)	-95	1.65 (0.18, 6.64)	-84	0.99 (0.03, 4.68)	NR

Risedronate vs Ibandronate	0.85 (0.60, 1.15)	70	1.29 (0.71, 2.19)	-36	NR	NR	NR	-25

Risedronate vs Raloxifene	0.84 (0.57, 1.15)	65	0.98 (0.61, 1.51)	622	0.79 (0.23, 1.96)	254	2.39 (0.05, 11.67)	-10

Strontium vs Alendronate	1.06 (0.81, 1.44)	-178	1.18 (0.78, 1.71)	-58	1.89 (0.61, 4.70)	-61	4.78 (0.14, 21.71)	NR

Strontium vs Denosumab	1.08 (0.75, 1.53)	-134	1.95 (1.20, 2.99)	-12	1.98 (0.44, 5.03)	-56	NR	-13

Strontium vs Etidronate	1.36 (0.65, 2.86)	-31	1.08 (0.51, 2.07)	-127	2.09 (0.20, 8.75)	-50	3.72 (0.05, 17.44)	NR

Strontium vs Ibandronate	0.95 (0.69, 1.34)	212	1.26 (0.70, 2.15)	-40	NR	NR	NR	-4

Strontium vs Raloxifene	0.94 (0.66, 1.34)	176	0.96 (0.60, 1.46)	243	1.03 (0.23, 2.66)	-1,789	10.85 (0.08, 41.99)	-6

Strontium vs Risedronate	1.12 (0.86, 1.57)	-90	0.99 (0.67, 1.43)	1,890	1.37 (0.44, 3.10)	-146	8.00 (0.15, 38.56)	-3

Strontium vs Teriparatide	1.38 (0.80, 2.35)	-29	1.99 (0.95, 3.66)	-11	4.92 (0.26, 24.44)	-15	19.69 (0.12, 80.47)	NR

Strontium vs Zoledronic Acid	1.17 (0.83, 1.66)	-64	2.17 (1.34, 3.34)	-10	1.93 (0.47, 4.98)	-59	NR	-49

Teriparatide vs Alendronate	0.77 (0.46, 1.31)	45	0.65 (0.31, 1.26)	28	1.35 (0.07, 5.71)	-154	1.69 (0.04, 8.09)	-102

Teriparatide vs Etidronate	0.98 (0.40, 2.30)	531	0.70 (0.39, 1.45)	24	1.54 (0.03, 9.01)	-100	1.33 (0.02, 6.65)	NR

Teriparatide vs Ibandronate	0.69 (0.40, 1.22)	33	0.53 (0.25, 0.98)	32	NR	NR	NR	-13

Teriparatide vs Raloxifene	0.68 (0.39, 1.19)	32	0.55 (0.26, 0.98)	21	0.76 (0.03, 3.27)	223	3.68 (0.02, 15.16)	-16

Teriparatide vs Risedronate	0.81 (0.49, 1.41)	55	0.55 (0.34, 1.04)	22	1.00 (0.05, 4.18)	NR	3.20 (0.04, 14.42)	NR

Zoledronic Acid vs Alendronate	0.91 (0.66, 1.30)	117	0.56 (0.34, 0.88)	22	1.24 (0.39, 3.16)	-225	NR	NR

Zoledronic Acid vs Etidronate	1.16 (0.55, 2.45)	-68	0.52 (0.23, 1.04)	20	1.38 (0.12, 5.70)	-142	NR	NR

Zoledronic Acid vs Ibandronate	0.82 (0.56, 1.19)	58	0.60 (0.31, 1.06)	25	NR	NR	NR	NR

Zoledronic Acid vs Raloxifene	0.81 (0.54, 1.19)	55	0.46 (0.26, 0.74)	18	0.68 (0.15, 1.78)	167	NR	NR

Zoledronic Acid vs Risedronate	0.96 (0.71, 1.41)	265	0.48 (0.29, 0.74)	18	0.91 (0.28, 2.07)	595	NR	NR

Zoledronic Acid vs Teriparatide	1.19 (0.68, 2.08)	-57	0.95 (0.45, 1.83)	216	3.11 (0.17, 16.12)	-26	NR	NR

NR: Not reported. Results are reported as Odds ratio.

For vertebral fractures, all drugs except etidronate had significant reductions in the odds of a fracture. The drugs with the highest probability of being most efficacious are teriparatide (0.30), zoledronic acid (0.40) and denosumab (0.20). However, the drugs with the highest effect size were also teriparatide (29.8), zoledronic acid (66.2) and denosumab (53.6) based on probabilities and effect size the three drugs are most efficacious. In addition, these 3 drugs also had the lowest number needed to treat versus the other drugs (Table 3). In the ITC head-to-head analysis, teriparatide had significant reduction in vertebral fracture versus ibandronate and raloxifene, while denosumab had significant reductions versus alendronate, raloxifene, and risedronate. Zoledronic acid had significant reductions versus alendronate, raloxifene, and risedronate (Table 3).

For hip fractures, only alendronate has a significant reduction in relative rate of fractures (OR = 0.59 (95%CrI: 0.29 to 0.99). The drugs that had the highest probability of most efficacious were teriparatide (0.44) and etidronate (0.19). The drugs with the highest effect size were alendronate (9.49) and risedronate (5.71). Based on probabilities and effect size it is unclear which drug might be ranked most efficacious out of the choices of teriparatide or alendronate. In the ITC head-to-head analysis, the relative efficacy of teriparatide versus alendronate was OR = 1.35 (95%CrI: 0.07, 5.71) which is a non-significant finding. There were no drugs that had a significant benefit for hip fractures versus the other drugs (Table 3).

For wrist fractures, there were no drugs that a significant protective effect versus placebo, although no wrist fracture data was available for denosumab, ibandronate or zoledronic acid. The drugs that had the highest probability of most efficacious were teriparatide (0.41) and risedronate (0.22). The drugs with the highest effect size were alendronate (1.80) and risedronate (1.37), although the magnitude of the effect size was considerably lower than for other fractures. Based on probabilities and effect size it is unclear which drug might be ranked most efficacious out of the choices of teriparatide or alendronate. In the ITC head-to-head analysis, the relative efficacy of teriparatide versus alendronate was OR = 1.69 (95%CrI: 0.04, 8.09) which is a non-significant finding. There were no drugs that had a significant benefit for wrist fractures versus the other drugs (Table 3).

### Assessing robustness: homogeneity and consistency of evidence

There was no difference between the estimates of the odds ratio and confidence or credibility intervals between the classical ITC software and the Bayesian WinBUGS ITC analysis.

**Figure 2:**
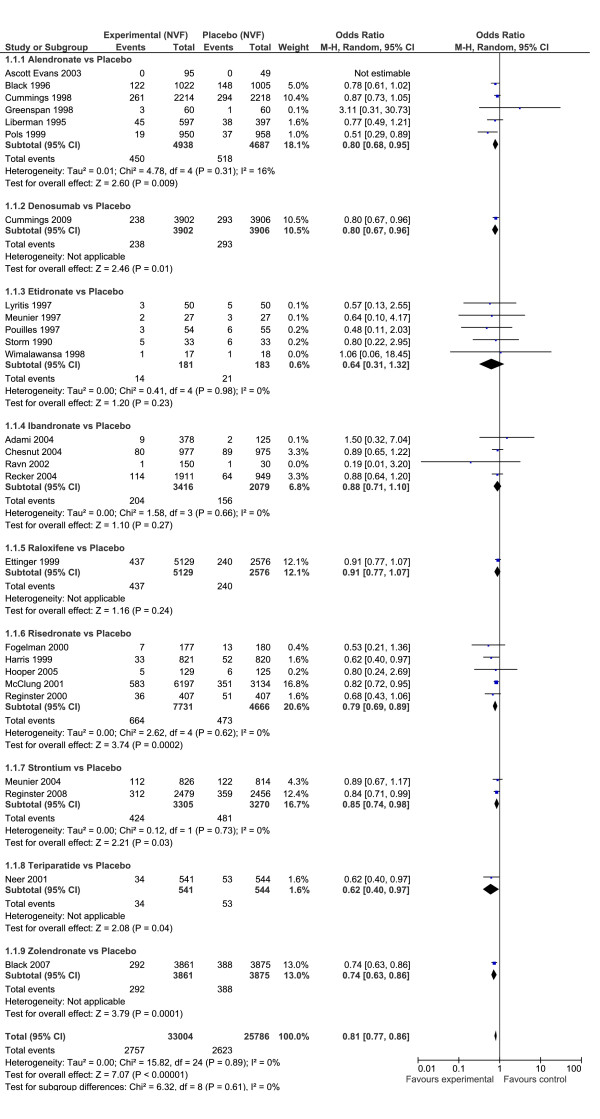
**Forest plot non vertebral fractures**. Odds ratio of non vertebral fractures for drugs versus placebo using Classical meta-analysis approach.

For non-vertebral fractures, the evidence was considered strong and free of bias because of low heterogeneity, and similarity of classical results to the Bayesian analysis. For non-vertebral fractures, the overall odds ratio across all drugs was OR = 0.81 (95% CI: 0.77, 0.86), (P < 0.01) indicating a protective effect of pharmacotherapy (Figure 2). There was no heterogeneity between types of drugs (I^2 ^= 0), although low heterogeneity (I^2 ^= 16%) existed for alendronate.

**Figure 3:**
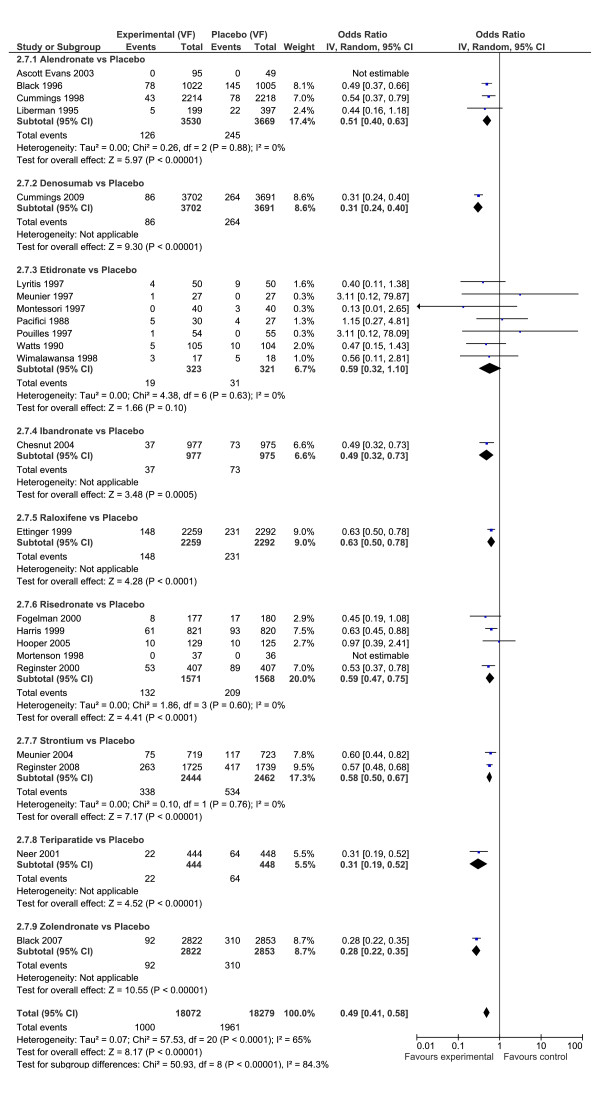
**Forest plot vertebral fractures**. Odds ratio of vertebral fractures for drugs versus placebo using Classical meta-analysis approach.

For vertebral fractures, the evidence is considered less strong than the evidence from non-vertebral fractures because of increasing heterogeneity (Figure 3), and the classical analysis having smaller confidence intervals than the Bayesian analysis. In the classical meta-analysis, the overall effect across all drugs was a protective effect in preventing vertebral fractures, OR = 0.49 (95% CI: 0.41, 0.58), and there was considerable heterogeneity across all drugs (I^2 ^= 84%), while there was no heterogeneity within drugs. All drugs except one provided significant predictive effects with the exception being etidronate, which produced a p-value of 0.10. Conversely, in the Bayesian analysis only risedronate and alendronate had significant odds ratios relative to placebo.

**Figure 4:**
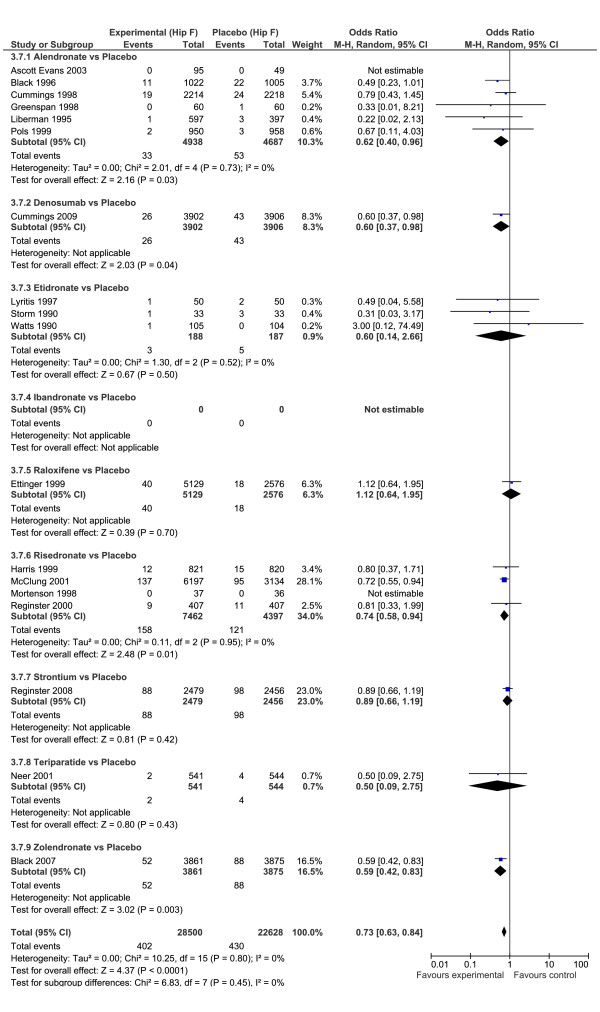
**Forest plot hip fractures**. Odds ratio of hip fractures for drugs versus placebo using Classical meta-analysis approach.

For hip fractures, the evidence is considered less strong than the evidence from non-vertebral fractures because of decreased confidence intervals in the classical analysis (Figure 4). For hip fractures, there was an overall protective effect against hip fracture for all drugs, OR = 0.73 (95% CI: 0.63, 0.84), and absence of heterogeneity (I^2 ^= 0%). Three drugs reported an independent statistical reduction in the rate of hip fracture, alendronate, OR = 0.62 (95% CI: 0.40, 0.96), denosumab OR = 0.60 (95% CI: 0.37, 0.98), and risedronate OR = 0.74 (95% CI: 0.58, 0.94). This is in contrast to the Bayesian analysis where only alendronate reported a significant reduction in the odds ratios for hip fracture.

**Figure 5:**
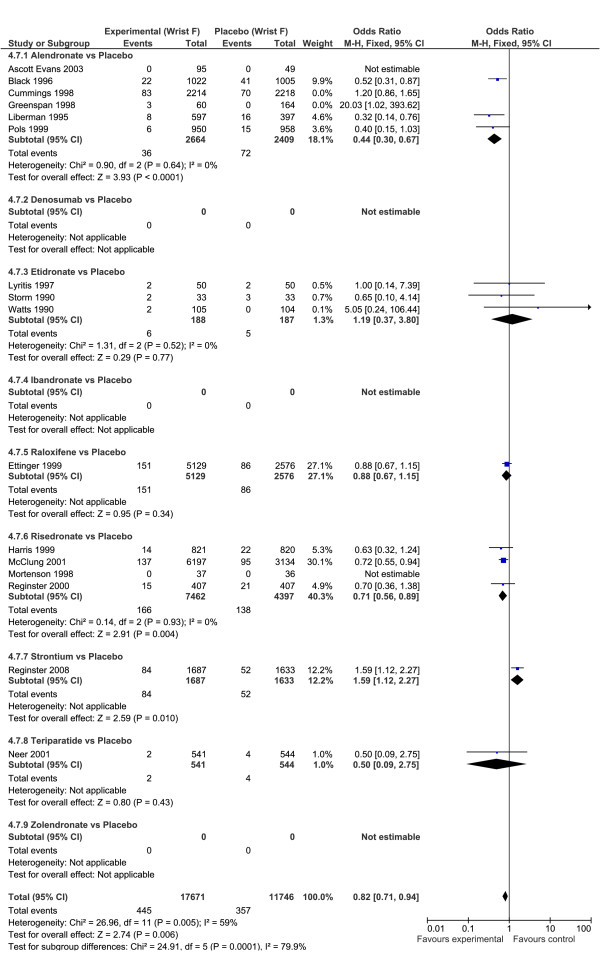
**Forest plot wrist fractures**. Odds ratio of wrist fractures for drugs versus placebo using Classical meta-analysis approach.

For wrist fractures, the evidence is considered weak because of increasing heterogeneity and differences in the classical versus Bayesian analysis when drugs were compared to placebo (Figure 5). For wrist fracture, there was not an overall protective effect OR = 0.88 (95% CI: 0.77, 1.01), and the heterogeneity was substantial (I^2 ^= 64%). The only drug that had a significant protective effect alone was risedronate OR = 0.71 (95% CI: 0.56, 0.89). The analysis of alendronate alone had considerable heterogeneity (I^2 ^= 79%). Removing Cummings and Greenspan to produce comparable ITC evidence reduced the heterogeneity to 0% and the odds ratio to OR = 0.44 (95% CI: 0.30 to 0.67) for alendronate versus placebo. Removing Cummings and Greenspan produced an overall odds ratio for all drugs OR = 0.82 (95% CI: 0.71, 0.94: I^2 ^= 59%).

### Adjustment for difference in baseline characteristics

The estimates of the relative efficacy with meta-regression for each drug versus placebo for each type of fracture were similar to the estimates of Bayesian analysis for odds ratios. Unfortunately, when baseline characteristics were added to the regression equation, there were not enough studies for the analysis and no estimate could be provided. This lack of result was created by the addition of the baseline characteristics age, BMD and rate of prior vertebral fractures that created multi-collinearity that was detected by exploded confidence intervals for each drug effect. When we ran the regression with only the top two drugs for each fracture along with adding in any of age, BMD or rates of prior vertebral fractures, the latter effects were significant while the drug effects was not significant. This suggests that the differences across studies in baseline characteristics contribute more to variation in odds ratio of fractures across studies than changes in the drugs.

## Discussion

The objective was to update the literature on the relative efficacy of different osteoporosis medications to prevent four types of osteoporosis-related fractures. Based on the combination of effect size and probability of being most efficacious, teriparatide zoledronic acid and denosumab are consistently ranked highest for reducing non-vertebral and vertebral fractures, the two most common types of fractures

Etidronate is also ranked high on probability of being most efficacious but there are reservations with this result. First, etidronate does not have a statistically significant odds ratio versus placebo for non-vertebral fracture, but was ranked highest for being efficacious. The higher ranking may be due to a wide confidence interval that covers a lower region of odds ratio creating a favourable relative result over that region of low odds ratio. This suggests a limitation with this analysis where a requirement may be that the odds ratio for different drugs should have similar widths. A second caution with the results for etidronate is that the trials were small resulting in small effect sizes and the trials were conducted prior to the year 2000. This suggests that there is a lack of current strong evidence for the efficacy of etidronate versus placebo. As a result of these two limitations, this analysis suggests that etidronate should not be considered among the most efficacious drugs based on current evidence.

In addition, the number needed to treat analysis that treating as few as 10 patients with teriparatide, zoledronic acid or denosumab will produce 1 less fracture than if the patients were on other drugs.

This work updates the most recent study for ITC analysis in osteoporosis medications which looked at vertebral, hip and nonvertebral nonhip fractures [13] for five drugs, zoledronic acid, alendronate, ibandronate, risedronate and etidronate. Based on that analysis zoledronic acid had a 0.79 probability of being the most efficacious for vertebral fractures. In our analysis, teriparatide (0.40) and etidronate (0.40) had the highest probability of being the most efficacious. In our analysis, we included more studies for etidronate, alendronate, and risedronate in addition to adding denosumab, raloxifene, strontium and teriparatide. Similarly, the earlier work reported that zoledronic acid had the highest probability of preventing hip fractures, while our analysis indicates the most efficacious drugs are teriparatide (0.44), and that zoledronic acid (0.11), etidronate (0.19), denosumab (0.12) and alendronate (0.10) could be the most efficacious treatment. One key difference between inclusions of different studies was that we analyzed wrist fractures specifically while the earlier work reported on nonvertebral nonhip fractures [13]. We report that risedronate does have a high probability of being most efficacious similar to earlier work but we estimated that teriparatide has the highest probability of preventing wrist fractures (0.44).

The other objective of this analysis was to compare the results across two statistical methods. The first method was based on Bayesian ITC analysis in WinBUGS, and the second method was the results from classical Bucher analysis with ITC specific software. The estimates differed only by the second decimal place when the results were statically significant. However, there are key differences in the interpretation of the results. Based on the classical analysis we generated confidence intervals around the odds ratio and provided a test of association. In the Bayesian analysis, we generated a posterior distribution of the credible intervals for the true values of the odds ratio. In this analysis these values are similar, indicating that the priors used in the analysis were uninformative.

The analysis is limited in that the results are based on ITC comparisons. However, a recent review of the results of DTC and ITC analysis, described that out of 44 meta-analysis that were available with studies for meta-analysis by ITC and studies for meta-analysis by DTC, the DTC was similar in all but 3 cases to the ITC estimates for the same drugs and outcomes [9]. Of the 3 cases where the results were statistically different, 2 cases had the relative clinical benefit in the same direction while the third had differences in dosage regime in the studies. This result was also reported by Bucher in 1997 [10] where the ITC results were similar in direction as the DTC estimates. In addition, Bucher and Song both reported that the magnitude of the ITC results was larger between comparators than DTC comparisons, and the level of significance between comparators was less in ITC than DTC. In our ITC analysis, non-significant differences were estimated between drugs but the true effect between drugs may be even smaller.

The other assessment of strength of evidence in the indirect comparisons beyond looking at different classical versus Bayesian analysis was to look at heterogeneity within drugs and across drugs. The heterogeneity between comparators and heterogeneity within one comparator was small, with the exception of alendronate for wrist fractures. This heterogeneity was explained by two studies [28,29] for wrist fractures. These studies did not contribute to heterogeneity in the meta-analysis of vertebral fractures and non-vertebral fractures. However, these two studies included the one study [28] that was the longest study with duration of 4 years with a low risk patients and the largest study for alendronate, while the other study [29] was small single centre study with duration of 4 years with low risk patients and the largest study.

The interpretation of the heterogeneity, although not a major feature in this analysis, is an important factor for ITC analysis. Increased heterogeneity can be caused by differences in inclusion criteria or study design such as length of follow-up. These are also important factors for consideration for analysis of DTC studies [20]. Three studies assessed the effect of patient characteristics to explain the level of heterogeneity in ITC analysis. In 2 studies [56,57] no baseline variables were significant while in the other study [58] the year of the study and baseline risk affected heterogeneity. Both of these factors may have also affected heterogeneity if the studies were randomized with an active comparator. In our analysis, we may not have enough power to detect the impact of baseline characteristics because of a low number of studies for each drug [22]. In addition, because of the high heterogeneity in the estimates of odds ratios for wrist fractures, the evidence for wrist fractures should be considered weak.

ITC is becoming a useful tool in the absence of DTC comparisons and increasing transparency of ITC analysis builds confidence for the evidence. In a review of 88 ITC analyses, many of the studies could have increased the believability of their results [9] but the missed elements would also concern DTC analysis. These include incomplete searches or not assessing heterogeneity within a comparator. In 40/88 analysis there was no specific searches for active comparison studies to allow the comparison to the ITC evidence. For osteoporosis, this search was conducted and we found no published meta-analysis of DTC evidence. In the future stronger evidence may come from head-to-head studies but this is unlikely, because based on this analysis differences between comparators are not significant and studies would require very large sample sizes. Alternatively, the treatment analysis could come for pooling patient level data to compare the effects directly but this is unlikely due to propriety, and this analysis would diminish the benefits of randomization.

## Conclusion

In light of the lack of DTC evidence, the ITC analysis of RPCTs may be the strongest evidence that will be available that answers the important clinical question of determining the most efficacious treatment for preventing fractures. In this analysis, teriparatide, zoledronic acid and denosumab have the highest probabilities of being most efficacious for non-vertebral and vertebral fractures, and having the greatest effect sizes. The estimates from indirect comparisons were robust to differences in methodology.

## Competing interests

Alexandra Papaioannou is or has been a consultant or on a speaker's bureau for Amgen, Aventis Pharma, Eli Lilly, Merck Frosst Canada, Novartis, Procter & Gamble Pharmaceuticals, Servier and Wyeth-Ayerst; she has conducted clinical trials for Eli Lilly, Merck Frosst Canada, Novartis, Procter & Gamble Pharmaceuticals and Sanofi-Aventis; and she has received unrestricted grants from Amgen, Eli Lilly, Merck Frosst Canada, Procter & Gamble Pharmaceuticals and Sanofi-Aventis. Jonathan Adachi has been a consultant or on a speaker's bureau for Amgen, Astra Zeneca, Eli Lilly, GlaxoSmithKline, Merck Frosst Canada, Novartis, Nycomed, Pfizer, Procter & Gamble Pharmaceuticals, Roche, Sanofi-Aventis, Servier, Wyeth-Ayerst and Bristol-Myers Squibb; he has conducted clinical trials for Amgen, Eli Lilly, GlaxoSmithKline, Merck Frosst Canada, Novartis, Pfizer, Procter & Gamble Pharmaceuticals, Sanofi-Aventis, Roche, Wyeth and Bristol-Myers Squibb. Lehana Thabane provides biostatistics consultation to GlaxoSmithKline for design and methodological issues. Eleanor Pullenayegum, Ron Goeree, Feng Xie and Robert Hopkins have no competing interests.

## Authors' contributions

RH carried out the literature review, data abstraction, statistical analysis and drafting of the manuscript. FX, EP, and LT provided guidance on the statistical analysis methodology. JD and AP provided guidance for the clinical interpretation of manuscript. All authors read, provided comments and approved the final manuscript.

## Pre-publication history

The pre-publication history for this paper can be accessed here:

http://www.biomedcentral.com/1471-2474/12/209/prepub

## Supplementary Material

Additional file 1 **Literature Search Strategy**. Strategy to find indirect treatment comparison controlled-trials for osteoporosis medications in Medline. Click here for file

Additional file 2 **WinBUGS ITC code for vertebral fractures**. WinBUGS software code and data to conduct ITC analysis for vertebral fractures. Click here for file
