# Supplementary material for: The relative efficacy of nine osteoporosis medications for reducing the rate of fractures in post-menopausal women
Source: BMC Musculoskelet Disord. 2011 Sep 26;12:209. doi: 10.1186/1471-2474-12-209 (PMC3196921; doi:10.1186/1471-2474-12-209)
Supplement: Additional file 1 — Literature Search Strategy. Strategy to find indirect treatment comparison controlled-trials for osteoporosis medications in Medline. [file 1471-2474-12-209-S1.DOCX]

Additional File 1: Literature Search Strategy (Medline)

1. exp Osteoporosis, Postmenopausal/

2. exp Postmenopause Osteoporosis/

3. ((postmenopaus* or post-menopaus*) adj1 (osteoporo* or bone loss or bone reduction)).ti,ab.

4. Fractures, Bone/pc

5. or/1-4

6. (84449-90-1 or 66376-36-1 or 105462-24-6).rn.

7. (raloxifene or evista).ti,ab.

8. (alendronate or fosamax or fosavance).ti,ab.

9. (risendronate or risedronate or actonel).ti,ab.

10. exp Raloxifene/

11. exp Alendronic Acid/

12. exp Alendronate/

13. exp Risedronic Acid/

14. or/6-13

15. Meta-Analysis.pt.

16. Meta-Analysis.sh. or exp Technology Assessment, Biomedical/

17. ((systematic$ adj (literature review$ or review$ or overview$)) or (methodologic$ adj (literature review$ or review$ or overview$))).ti,ab.

18. ((quantitative adj (review$ or overview$ or synthes$)) or (research adj (integration$ or overview$))).ti,ab.

19. ((integrative adj2 (review$ or overview$)) or (collaborative adj (review$ or overview$)) or pool$ analy$).ti,ab.

20. (data synthes$ or data extraction$ or data abstraction$).ti,ab.

21. (handsearch$ or hand search$).ti,ab.

22. (meta analy$ or metaanaly$ or met analy$ or metanaly$ or health technology assessment$ or HTA or HTAs or biomedical technology assessment$ or bio-medical technology assessment$).ti,ab.

23. (meta regression$ or metaregression$ or mega regression$).ti,ab.

24. (Meta Analysis or Systematic Review or Biomedical Technology Assessment).sh.

25. or/15-24

26. 5 and 14 and 25
