# Supplementary material for: The relative efficacy of nine osteoporosis medications for reducing the rate of fractures in post-menopausal women
Source: BMC Musculoskelet Disord. 2011 Sep 26;12:209. doi: 10.1186/1471-2474-12-209 (PMC3196921; doi:10.1186/1471-2474-12-209)
Supplement: Additional file 2 — WinBUGS ITC code for vertebral fractures. WinBUGS software code and data to conduct ITC analysis for vertebral fractures. [file 1471-2474-12-209-S2.DOC]

Additional file 2. WinBUGS code and data

# vertebral fracture

model {

for (i in 1:N) {logit(p[i])<-mu[s[i]]+delta[i] * (1-equals(t[i],b[i]))

r[i]~dbin(p[i],n[i])

delta[i]~dnorm(md[i],tau)

md[i]<-d[t[i]] - d[b[i]] }

for (j in 1:NS) { mu[j]~dnorm(0, .001) }

d[1]<-0

for (k in 2:NT) { d[k] ~dnorm(0,.001) }

sd~dunif(0,2)

tau<- 1/pow(sd,2)

for (i in 1:N) {mu1[i]<-mu[s[i]]*equals(t[i],1) }

for (k in 1:NT) {logit(T[k]) <- sum(mu1[])/23+d[k] }

# ranking and probability { treatment is most effective}

for (k in 1:NT) {rk[k] <-rank(T[],k)

best[k]<-equals(rk[k],1)}

#all pairwise odds ratios

for (c in 1:(NT-1)) {for (k in (c+1):NT) {or[c,k] <- exp(d[k]-d[c])}}

}

# s[] indicates study

# t[] treatment

# r[]numerator

# n[]denominator

# b[] comparator treatment for that trial , b[i]<=t[i] ( =1 if all placebo based )

# treatment

# 1 placebo

# 2 alendronate

# 3 etidronate

# 4 ibandronate

# 5 raloxifene

# 6 Risedronate

# 7 Teriparatide

# 8 ZA

# 9 Denosumab

# 10 Strontium

Data

list(N=46, NS=23, NT=10)

s[] t[] r[] n[] b[]

1 1 0 49 1

1 2 0 95 1

2 1 145 1005 1

2 2 78 1022 1

3 1 78 2218 1

3 2 43 2214 1

4 1 22 397 1

4 2 5 199 1

5 1 9 50 1

5 3 4 50 1

6 1 0 27 1

6 3 1 27 1

7 1 3 40 1

7 3 0 40 1

8 1 4 27 1

8 3 5 30 1

9 1 0 55 1

9 3 1 54 1

10 1 10 104 1

10 3 5 105 1

11 1 5 18 1

11 3 3 17 1

12 1 73 975 1

12 4 37 977 1

13 1 231 2292 1

13 5 148 2259 1

14 1 17 180 1

14 6 8 177 1

15 1 93 820 1

15 6 61 821 1

16 1 10 125 1

16 6 10 129 1

17 1 0 36 1

17 6 0 37 1

18 1 89 407 1

18 6 53 407 1

19 1 64 448 1

19 7 22 444 1

20 1 310 2853 1

20 8 92 2822 1

21 1 264 3691 1

21 9 86 3702 1

22 1 417 1739 1

22 10 263 1725 1

23 1 117 723 1

23 10 75 719 1

END
